# The Impact of the COVID-19 Lockdown on the Cognitive Functions in Persons with Intellectual and Developmental Disabilities

**DOI:** 10.3390/ijerph192315511

**Published:** 2022-11-23

**Authors:** Raquel Braga, Elena Felipe-Castaño

**Affiliations:** 1 Association Juan XXIII, 36003 Pontevedra, Spain; 2 Personality, Assessment, and Psychological Intervention, University of Extremadura, 10073 Cáceres, Spain

**Keywords:** intellectual disability, lockdown, CAMCOG, follow-up study

## Abstract

The main objective of the research was to compare the cognitive functioning of a sample of persons with IDD (Intellectual and Developmental Disabilities) before the pandemic (2019) and after the pandemic (2020 and 2021), and to analyse the impact according to age and level of IDD impairment. The participants were 92 persons with IDD, of whom 43 were female (46.7%). The mean age in 2019 was 47.07 years (SD = 6.78). All the participants were living with family members. The CAMCOG-DS test from CAMDEX was used to assess the cognitive functions. The results indicate a worsening in cognitive functions (attention–concentration, abstract thought, language, and praxis) after lockdown, in both the total group of participants and the mild–moderate impairment group, and in both age groups. In the severely affected group, we found an improvement in the cognitive functions assessed after lockdown. These results are similar to those found in people with dementia and in the general ageing population. Results were discussed in relation to the consequences of isolation in people with IDD, as well as providing guidelines for future pandemic situations.

## 1. Introduction

The COVID-19 pandemic and compulsory lockdown have affected the wellbeing of the entire population, particularly that of persons needing special support, such as those with intellectual and developmental disabilities (IDDs).

Persons with IDDs are more vulnerable to changes in their routine and daily activities, as they require daily, face-to-face attention and support services oriented towards their education, treatment, rehabilitation, or occupational work [1]. This attention and services were interrupted or closed during lockdown, bringing about a deterioration in their emotional wellbeing and their occupational and leisure activities [2], as well as a reduction in their physical activity and an increased sedentary lifestyle [3,4]. In general, their social isolation also increased, due to both the lockdown and the restrictions imposed on interpersonal contact [5].

In the general population, the social isolation associated with the pandemic was related to cognitive impairment; even relatively short periods of social isolation could have a negative effect on the cognitive skills and the executive functions [6]. In elderly people, loneliness caused by social isolation has also been associated with the impairment of cognitive functions [7]. Persons with IDD are generally more isolated than their peers without IDD, so it is probable that the impact of lockdown also had negative consequences for their cognitive functions.

The quality of life of the professionals in direct care and family members was also negatively affected by the restrictions [8]. For the professionals involved in direct care, the lockdown had a generalised impact, both emotional, related to the events experienced, and cognitive, with respect to the challenges affecting their work, in practical ways on carrying out their work duties during the pandemic and, professionally, concerning their experiences alongside other professionals [9].

In other population groups, such as persons diagnosed with dementia, direct neurological effects have been found from being infected by the virus causing COVID-19, as well as indirect effects [10]. One of the indirect effects was described as a cognitive affectation, especially concerning concentration, orientation, memory, and communication [11]. In addition, lockdown exacerbated the neuropsychiatric symptoms already present in persons suffering from dementia residing in institutions [12], in which new symptoms were detected such as apathy, anxiety, and disrupted motor functions [13].

The impact of lockdown on the physical health and wellbeing of the general population is being widely studied [14,15,16]. In persons with IDD, the reviewed studies refer to the negative effects, but there are very few empirical studies concerning this impact and the consequences of lockdown [17], particularly as far as the cognitive functioning is concerned. The main objective of this work is thus to compare the cognitive functioning of a sample of persons with IDD before (year 2019) and after (years 2020 and 2021) the pandemic, as well as to make an analysis of this impact according to age and the severity of the IDD.

As there are no available prior works concerning the impact of similar situations on the cognitive functions of persons with IDD, we use as a basis the conclusions of the studies carried out on persons with dementia [11]. According to these studies, we pose the hypothesis that lockdown has a negative impact, resulting in the worsening of the cognitive functions as compared with the prior functioning. Furthermore, we consider that this impact will be different for the participants depending on the severity of the IDD (light–moderate and serious–severe) and the age group (up to 45 years of age and over 46 years of age).

## 2. Materials and Methods

A prospective or longitudinal intra-group design of repeated measures [18] was carried out before and after lockdown (2019, 2020, and 2021). In our country, lockdown was extended from 14 March to 3 May. During compulsory confinement, going outside was forbidden, and only allowed for work or to care for elderly or dependent persons, with restrictions on public transportation and shopping.

### 2.1. Participants

The selection of the participants was performed using intentional (non-probabilistic) sampling among persons belonging to an association of persons with intellectual disabilities. There were 92 participants with intellectual and developmental disabilities (IDD), of whom 43 were female (46.7%). The average age of all the participants in 2019 was 47.07 (SD = 6.78). The exclusion criterion was set as having a diagnosed neurological disorder in particular, dementia, cognitive impairment, and long-standing epilepsy, at the start of the study and, as inclusion criteria, being between 35 and 70 years of age and consenting to participation in the research.

All the selected participants were living at home and were attending the specialised attention and occupational therapy services on a daily basis in centres belonging to the association. Table 1 sets out other sociodemographic characteristics of the participants. The aetiology of the most frequent intellectual disability was the non-specified and the degree of severity was the light–moderate. The grade of severity of participants was assessed by community mental health services. No differences were found in the percentages of males and females according to the aetiology of the IDD (χ^2^ = 0.470; *gl* = 3; *p* = 0.925), the degree of severity (χ^2^ = 3.527; *gl* = 2; *p* = 0.171), or the age group (χ^2^ = 0.186; *gl* = 1; *p* = 0.666). Nor did we find differences in the percentage of participants with respect to the age group or the severity of the IDD (χ^2^ = 3.374; *gl* = 1; *p* = 0.066). Of all the participants, only two stated having been infected by COVID-19 (2%), a percentage close to that obtained by the participants in the same type of residence [19].

### 2.2. Instruments

The instrument used for the research was the Cambridge Examination for Mental Disorders of Older People with Down Syndrome and others with Intellectual Disabilities (CAMDEX-DS) [20]. The Spanish adaptation is by Esteba-Castillo et al. [21].

The CAMDEX-DS aims to evaluate the most frequent forms of dementia, as well as other mental and physical disorders in older persons with Down syndrome or an intellectual disability with a different aetiology. It is made up of three parts: the inventory, a guide for clinical diagnosis, and a guide for interventions following the diagnosis. The inventory is made up of two sections: a structured interview with an informant (family member or carer), which includes questions concerning changes in the functional ability or the behaviour that such changes may have brought about; an evaluation of the patient that includes a cognitive exam (CAMCOG-DS) to make a brief neuropsychological evaluation, carried out directly with the patient.

For this research, although the entire inventory was administered, only part III, the brief cognitive exam (CAMCOG-DS) was used, which is included in Section 2 (evaluation of the patient). The CAMCOG DS evaluates the specific cognitive deficits in the operative diagnostic criteria for dementia, such as amnesic deficits, aphasia, apraxia, and agnosia (executive function). The scales are: orientation, language (comprehension and expression), memory (remote and recent new learning), attention–concentration, praxis (drawing complex figures and carrying out complex tasks), abstract thought, and perception, as well as a final total score. The lowest scores in the CAMCOG-DS suppose a worsening of the cognitive functions and a greater risk of cognitive deterioration, as long as there is a prior baseline score [21].

The internal consistency values measured using Cronbach’s alpha can be seen in Table 2. The values are good, except in the scale of perception, and similar to those of the Spanish adaptation [21].

### 2.3. Procedure

The application of the CAMDEX DS forms part of the annual evaluation protocol used in the centre where the participants attend for treatment on a daily basis. The test was administered individually, by a psychologist of the centre, complying with the requirements demanded for its administration (knowing the person for at least six months and formation on applying the scale). The CAMCOG-DS was applied to the person with IDD him/herself. The evaluation was carried out in a single session lasting between 40 and 60 min, taking into account the aspects of psychological evaluations for persons with IDD. The results obtained were included in the clinical records of the person being evaluated.

The study was approved by the Bioethics Committee of the Juan XXIII Association (12 November 2019). The research followed the recommendations of the Declaration of Helsinki, with its revisions, and informed consent was requested in writing from each participant and/or his/her legal tutor, depending on the case.

### 2.4. Data Analysis

The Kolmogorov–Smirnov (K-S) test was used to analyse the standard fit of the research variables and they were found to be non-normally distributed (*p* < 0.05). We therefore decided to use non-parametric tests for data analysis. We carried out descriptive analyses, central tendency, and dispersion (mean, standard deviation, median, range, frequencies, and percentages). To analyse the differences in the frequency of responses between groups, we used contingency tables and the χ^2^ test. The differences between the scores in the CAMCOG-DS scales over the two and three years (related samples) were analysed using the Z Range test with the Wilcoxon sign and Friedman’s χ^2^ test, respectively. Cohen’s d [22] was used to estimate the size of the effect of the differences.

The statistical analyses were carried out using the SPSS 29 statistical package. A level of significance of 95% (*p* < 0.05) was assumed in all the analyses.

## 3. Results

We first of all set out the results obtained with the total group of participants, and then, the results obtained broken down into the data for the groups established according to the severity of the IDD light–moderate and serious–severe, following the prior diagnosis, and the age groups up to 45 years and 46 or over, taking into account that the age of 45 is considered to be the cut-off point for entering old age for persons with IDD [23].

No statistically significant differences were found in the scores of the CAMCOG-DS scales, over the three-year period, in the total number of participants. Table 2 shows the descriptive statistics of the scores for each year evaluated in the CAMCOG-DS scales for the total number of participants. A decrease can be seen in the scores over the three evaluations, except in the orientation scale, in which there is an increase, although these differences are not statistically significant. Analysing the differences, year by year, statistically significant differences are only found in the scale attention–concentration (*Z* = −2.776; *p* = 0.008) before and after lockdown (2019–2020), in the sense of a lower score in 2020, which supposes a worse performance in attention–concentration once lockdown had finished.

In the group of participants with light–moderate severity, we found significant differences between the three years in the scale of abstract thought (χ^2^ = 6.750; *gl* = 2; *p* = 0.034) in the sense of a lower score after lockdown, i.e., a deterioration in abstract thought for both 2020 and 2021.

If the comparison is carried out year by year, significant differences were found in the light–moderate group between 2019 and 2020 in the scales attention–concentration and abstract thought of the CAMCOG-DS, in the sense of a lower score in 2020, although with a small effect size. Between 2019 and 2021, we found significant differences in the scales of language, attention–concentration, praxis, and abstract thought, in the sense of a lower score in 2021 (see Table 3), with a moderate effect size. The deterioration continues in abstract thought and attention and, in 2021, there was also an effect on language and praxis.

In the serious–severe group, we found differences in the scales of orientation (χ^2^ = 16.441; *gl* = 2; *p* < 0.000), memory (χ^2^ = 8225; *gl* = 2; *p* = 0.016), attention–concentration (χ^2^ = 9.656; *gl* = 2; *p* = 0.008), and the total score of the CAMCOG (χ^2^ = 13.368; *gl* = 2; *p* < 0.001), in the sense of a higher score following lockdown, which supposes an improvement in these cognitive functions (see Table 4).

As for the year-by-year differences, differences were found between 2019 and 2021 in the scales of orientation, language, memory, attention–concentration, abstract thought, and the total score of the CAMCOG-DS, in the sense of a higher score in 2021. The size of the effect is moderate, except in orientation, where it is large. This result indicates a better cognitive state in the 2021 evaluation compared with that of 2019. No differences were found between 2019 and 2020.

No significant differences were found in the scales of the CAMCOG-DS between the three years in the group of those 45 years of age and under. Comparing year by year, between 2019 and 2020 (see Table 5), differences were found in the scales of praxis and the total score of the CAMCOG-DS, in the sense of higher scores in 2019. This result supposes a worse functioning in praxis and general cognitive performance in the group of those up to 45 years of age following lockdown, although the size of the effect is small.

Statistically significant differences were found in the group of participants aged 46 and over between the three years in the scales of orientation (χ^2^ = 6.170; *p* = 0.046) and perception (χ^2^ = 6.687; *p* = 0.035).

As for the comparisons year by year, in those aged 46 and over (see Table 6), differences were found in the scales of attention–concentration and perception of the CAMCOG-DS, in the sense of a higher score in 2019 in comparison to 2020, which supposes a worse functioning following the lockdown. As for the differences with 2021, they were found in the scale of perception of the CAMCOG-DS, also in the sense of a higher score in 2019. The size of the effect was small in all of the above. This result supposes that the orientation and perception of those aged 46 and over have been affected during lockdown.

## 4. Discussion

The main objective of this research was to describe and compare the cognitive functioning of a sample of persons with IDD before and after lockdown, as well as to analyse their cognitive state according to the severity of the IDD and their age.

In the total number of participants, the studies indicate that their cognitive state was affected, with a worsening of their cognitive functions, but less significant than perhaps expected. The cognitive function in which the greatest impact was observed was in attention–concentration.

If we take into account the degree of severity of the IDD and the age of the participants, the results found are different. Thus, the light–moderate group showed differences in the cognitive exam in five of the seven scales, with a better functioning before the pandemic in attention–concentration, abstract thought, language, and praxis. As for the seriously affected group, orientation, language, memory, attention–concentration, abstract thought, and the total score were all affected, but in the sense of an improvement in the score in the year following the lockdown.

These results indicate that the lockdown has resulted in a cognitive deterioration in persons with light–moderate IDD, while there were no changes in those with serious IDD, and even a year after the lockdown, there has been an improvement in their cognitive functioning. In the case of the light–moderate group, the results point in the direction of the initial hypothesis, coinciding with the studies carried out both on persons with dementia [13,24] and older healthy persons [25]. The loss of structured activities due to lockdown, with the interruption of external support services, the reduction in social and interpersonal contacts and leisure activities, among others, have had a negative impact on their prior functioning, which was still negative a year after the end of lockdown.

As for those with a serious affectation, other aspects need to be considered to explain this result, since they contradict the proposed hypothesis and the results found in research into persons with dementia [13,24]. That the impact of lockdown on the cognitive functions was not negative in the seriously affected group may be explained by such aspects as less disruption to routines, and interpersonal and leisure activities, which were not limited to such an extent during lockdown in comparison with persons with a light–moderate affectation [26,27,28]. Another possibility would be a return to normal of the services may have favoured their cognitive functioning in a positive way, overcoming even the possible negative impact of lockdown. In this sense, they have been able to benefit from the changes implemented in the care services as a consequence of the obligatory prevention measures, including working with smaller groups and greater support. Furthermore, but without underestimating the importance of social inclusion, the reduction in seating capacity in public areas, sporting activities, rest areas, restaurants, etc, could have favoured a climate of tranquillity, with a greater control over the environment, in both time and space, which may have favoured their orientation and general cognitive functioning.

On the other hand, these aspects may not have favoured so much those persons with light–moderate IDD, since the situation they returned to was very different to that which they were accustomed, with a new structure for services and new demands from the environment. The difficulties described in the adaptive behaviour and the coping strategies [29] may have interfered with the recuperation of the cognitive functions following the lockdown.

Furthermore, all of our participants lived with family during the lockdown, persons with whom they had a stable relationship providing support. This could have been an advantage, enabling them to perceive themselves as being less isolated, and thus able to be more positive, especially those with a serious affectation, in comparison with those persons in other living circumstances, such as residences and institutions, in which the isolation and restrictions imposed on social contact affected the users [19]. On the other hand, living with family could also justify a loss of such functions as praxis, due to overprotection in the activities of daily life, as the family carry out activities, such as cleaning or tidying up for them, that in the occupational centre are being performed by the patients themselves.

As for the results according to the age group, we confirmed the proposed hypothesis, since we found deterioration in both groups of the cognitive functions, although in different cognitive areas: in praxis and the total CAMCOG-DS score for 45 and under; and in attention–concentration and perception in the 46 year olds and over. It should be taken into account that, in persons with IDD, it is considered that old age starts at approximately 45 years of age, earlier than in the general population, always keeping in mind that ageing is a specific phenomenon of each individual, where the start will be marked by the presence of deterioration [23]. Neither should it be forgotten that even healthy ageing can present abnormalities in the cognitive and mental skills, as well as in the functional skills [30].

## 5. Conclusions

In conclusion, in our participants, the obligatory lockdown had a negative effect on the cognitive functions, particularly on persons with a light–moderate IDD, an impact that has been sustained up to a year after lockdown. As for age, both groups were affected, although in different functions: in praxis for those 45 and under, and in attention–concentration and perception in those 46 and over.

Taking into account the exceptional circumstances of the situation being lived through, it is necessary to know the consequences of the situation in order to be able to implement programmes and to avoid the negative impact in similar situations. Knowing the impact of the lockdown and the isolation on the health and wellbeing of persons with IDD is crucial, as it can provide us with insights into the risks and benefits of the measures taken, as well as providing knowledge to help us plan the provision of care in future pandemics [11].

In this sense, the pandemic has brought about modifications in the services offered to persons with IDD, as well as opportunities to improve and develop programmes, that could be implemented in the future. The provision of information concerning COVID-19 is also important, as are the protective measures taken to avoid contagion, including the production of material to raise awareness of the expansion of the virus and guaranteeing the maintenance of personal relationships through online means [31]. To this, we would also add the development of virtual methodologies, which can allow treatments, education, and social interaction to be maintained.

The study presented here has limitations that have been taken into consideration when interpreting the results. One is the small sample of participants, which was even further reduced on dividing the data in some of the analyses. All the participants lived with their families, so the conclusions cannot be generalised to other living situations, such as those living in residences or institutions. It was not possible to include all the participants in all the evaluation years due to differing personal situations that made the evaluations inviable. This is one of the risks of longitudinal designs, together with the loss of subjects [32]. Even though this has been taken into account when carrying out the statistical analyses, it must also be considered when generalising the results. Finally, the instrument used has some limitations that may have affected the data collected; thus, the contents of some questions should therefore be updated, and the understanding of others improved [33]. In the case of our study, the perception scale of the CAMCOG-DS presents a low internal consistency, which means we should consider the results of this scale with caution and look at its content in detail so, as to improve its construction and thus its reliability [34].

In future works, we will continue to analyse the long-term consequences of the lockdown through the evaluations being carried out, as in many respects this impact remains unknown. We will continue to register evaluations and to analyse the data so as to be able to extract conclusions over the long term. On the other hand, we shall also widen the research to include participants with different living situations so as to be able to compare the associated impact. Nevertheless, this aspect will not be possible if evaluations prior to the pandemic do not exist.

## Figures and Tables

**Table 1:** Sociodemographic characteristics of the participants.

	M	SD	Min	Max	*n*	%
Age of total participants	47.07	6.78	35	65		
Male	47.18	7.49	35	65		
Female	46.93	6.96	39	63		
Groups according to age						
≤45 years					45	48.9
≥46 years					47	51.1
Main occupation						
Special employment centre					1	1.1
Occupational therapy service					21	22.8
Specialised care centre					70	76.1
Aetiology of the IDD						
Not specified					37	40.2
Down syndrome					31	33.7
Others					24	26.1
Severity of the IDD						
Light–moderate					61	66.3
Serious–severe					31	33.7

**Table 2:** Descriptive statistics and value of Cronbach’s α. CAMCOG-DS scales.

	2019				2020				2021			
	M(SD)	Mdn	AR	α	M(SD)	Mdn	AR	α	M(SD)	Mdn	AR	α
Orientation	8.84(3.02)	9	1.84	0.757	9.07(2.93)	10	2.03	0.778	9.24(3.35)	10	2.14	0.810
Language	18.64(5.00)	19	2.03	0.819	18.17(5.00)	18	1.98	0.834	17.77(5.54)	18	1.99	0.849
Memory	15.75(6.56)	16	1.97	0.778	15.52(6.57)	16	1.91	0.749	15.25(6.89)	16	2.13	0.763
Attention	6.21(2.70)	7	2.05	0.757	5.68(2.51)	6	1.82	0.761	5.77(2.72)	6	2.13	0.770
Praxis	12.60(3.78)	13	2.04	0.780	12.28(3.38)	13	1.96	0.706	11.59(4.20)	12	2.00	0.794
Abstract thought	2.38(2.28)	2	2.09	0.805	2.10(2.23)	2	1.97	0.785	1.97(2.17)	2	1.93	0.789
Perception	4.89(1.68)	5	2.15	0.275	4.65(1.71)	5	2.15	0.331	4.69(1.87)	5	2.03	0.541
Total score	68.67(21.48)	70	2.00	0.943	67.50(20.36)	68	1.80	0.940	66.28(22.01)	67	2.20	0.944

NOTE: AR = Average range; α = Cronbach’s alpha.

**Table 3:** Light–moderate group (*n* = 61). 2019–2020/2019–2021. Wilcoxon Z Range test for related samples.

Years	2019			2020			Z	Sig.	Cohen’s d
	M(SD)	Mdn	Range	M(SD)	Mdn	Range			
Attention–concentration	7.21(2.27)	8	12	6.43(2.37)	7.5	9	−2.431	0.015	0.336
Abstract thought	3.12(2.25)	4	6	2.61(2.32)	2	6	−2.061	0.039	0.223
**Years**	**2019**			**2021**			**Z**	**Sig.**	**Cohen’s d**
	**M(SD)**	**Mdn**	**Range**	**M(SD)**	**Mdn**	**Range**			
Language	20.58(3.98)	21	16	18.45(5.93)	20	26	−2.088	0.037	0.422
Attention–concentration	7.21(2.27)	8	12	6.00(2.72)	7	9	−2.378	0.017	0.483
Praxis	13.97(2.90)	14.5	11	12.30(3.80)	13	17	−2.029	0.043	0.494
Abstract thought	3.12(2.25)	4	6	2.09(2.14)	2	6	−2.612	0.009	0.469

**Table 4:** Serious–severe group (*n* = 31) 2019–2021. Wilcoxon Z Range test for related samples.

	2019			2021			Z	Sig.	Cohen’s d
	M(SD)	Mdn	Range	M(SD)	Mdn	Range			
Orientation	6.66(2.75)	7	10	9.39(3.93)	10	16	−2.956	0.003	0.804
Language	14.68(4.55)	15	19	16.50(4.56)	17	17	−2.245	0.025	0.399
Memory	11.00(5.14)	10	18	13.46(5.44)	14.5	19	−2.147	0.032	0.464
Attention– concentration	4.24(2.40)	3	8	5.35(2.72)	6	9	−2.485	0.013	0.432
Abstract thought	0.90(1.47)	0	4	1.75(2.27)	0	6	−2.183	0.029	0.444
Total score	50.41(13.17)	44	61	61.07(19.92)	61	74	−3.190	0.001	0.631

**Table 5:** Comparison 2019–2020. Age group up to 45 inclusive (*n* = 45). Wilcoxon Z Range test for related samples.

	2019			2020			Z	Sig.	Cohen’s d
	M(SD)	Mdn	Range	M(SD)	Mdn	Range			
Praxis	13.39(3.48)	14	13	12.79(3.38)	13	11	−2.422	0.015	0.174
Total score	72.46(19.68)	75	72	69.72(18.81)	68	75	−2.171	0.030	0.142

**Table 6:** Comparison 2019–2020/2019–2021. Age group ≥ 46 years (*n* = 47). Wilcoxon Z Range test for related samples.

Years	2019			2020			Z	Sig.	Cohen’s d
	M(DT)	Mdn	Range	M(DT)	Mdn	Range			
Attention–concentration	5.80(2.81)	6	12	5.31(2.69)	6	9	−2.435	0.015	0.178
Perception	5.11(1.77)	5	7	5.31(2.69)	6	9	−2.724	0.006	0.087
**Years**	**2019**			**2021**			**Z**	**Sig.**	**Cohen’s d**
	**M(DT)**	**Mdn**	**Range**	**M(DT)**	**Mdn**	**Range**			
Perception	5.11(1.77)	5	7	4.56(1.93)	5	8	−1.970	0.049	0.297

## Data Availability

Not applicable.

## References

[1] World Health Organization Disability Considerations during the COVID-19 Outbreak. https://apps.who.int/iris/bitstream/handle/10665/332015/WHO-2019-nCov-Disability-2020.1-tur.pdf.

[2] Amor A.M., Navas P., Verdugo M., Crespo M. (2021). Perceptions of people with intellectual and developmental disabilities about COVID-19 in Spain: A cross-sectional study. J. Intellect. Disabil. Res..

[3] Gutiérrez-Cruz C., Muñoz-López S., Román-Espinaco A. (2021). Effect of COVID-19 lockdown on body composition of residents with intellectual disability. Span. J. Disabil. Stud..

[4] Theis N., Campbell N., De Leeuw J., Owen M., Schenke K.C. (2021). The effects of COVID-19 restrictions on physical activity and mental health of children and young adults with physical and/or intellectual disabilities. Disabil. Heal. J..

[5] Courtenay K., Perera B. (2020). COVID-19 and people with intellectual disability: Impacts of a pandemic. Ir. J. Psychol. Med..

[6] Ingram J., Hand C.J., Maciejewski G. (2021). Social isolation during COVID -19 lockdown impairs cognitive function. Appl. Cogn. Psychol..

[7] Lithander F.E., Neumann S., Tenison E., Lloyd K., Welsh T.J., Rodrigues J.C.L., Higgins J.P.T., Scourfield L., Christensen H., Haunton V.J. (2020). COVID-19 in older people: A rapid clinical review. Age Ageing.

[8] Murray G.C., McKenzie K., Martin R., Murray A. (2021). The impact of COVID-19 restrictions in the United Kingdom on the positive behavioural support of people with an intellectual disability. Br. J. Learn. Disabil..

[9] Sheerin F., Allen A.P., Fallon M., McCallion P., McCarron M., Mulryan N., Chen Y. (2022). Staff mental health while providing care to people with intellectual disability during the COVID-19 pandemic. Br. J. Learn. Disabil..

[10] Iodice F., Cassano V., Rossini P.M. (2021). Direct and indirect neurological, cognitive, and behavioral effects of COVID-19 on the healthy elderly, mild-cognitive-impairment, and Alzheimer’s disease populations. Neurol. Sci..

[11] Suárez-González A., Rajagopalan J., Livingston G., Alladi S. (2021). The effect of COVID-19 isolation measures on the cognition and mental health of people living with dementia: A rapid systematic review of one year of quantitative evidence. eClinicalMedicine.

[12] Numbers K., Brodaty H. (2021). The effects of the COVID-19 pandemic on people with dementia. Nat. Rev. Neurol..

[13] Lara B.B., Carnes A., Dakterzada F., Benítez I.D., Piñol-Ripoll G. (2020). Neuropsychiatric symptoms and quality of life in Spanish patients with Alzheimer’s disease during the COVID-19 lockdown. Eur. J. Neurol..

[14] Chiesa V., Antony G., Wismar M., Rechel B. (2021). COVID-19 pandemic: Health impact of staying at home, social distancing and ‘lockdown’ measures—a systematic review of systematic reviews. J. Public Heal..

[15] Lippi G., Henry B.M., Bovo C., Sanchis-Gomar F. (2019). Health risks and potential remedies during prolonged lockdowns for coronavirus disease 2019 (COVID-19). Diagnosis.

[16] Patti A., Giustino V., Figlioli F., Miceli M., Barca M., Drid P., Palma A., Bianco A. (2022). The role of school physical education on adolescents’ fitness levels during the pandemic period from COVID-19: An observational study of the Italian scientific high school—section sport and physical activity. Front. Public Heal..

[17] Embregts P.J.C.M., Nijs S.L.P., van Oorsouw W.M.W.J. (2021). Impact of infection outbreak on people with intellectual disabilities: A scoping review. J. Intellect. Dev. Disabil..

[18] Ato García M., Vallejo Seco G. (2015). Diseños de Investigación en Psicología.

[19] Rodríguez C.C., García I.Y., Bargues S., Martín R.M. (2022). ¿Cómo vivieron las personas con discapacidad la crisis de la COVID-19? El caso de las personas apoyadas por entidades tutelares en España [How Did People with Disabilities Experience the COVID-19 Crisis? Situation of People Supported by Guardianship Organizations in Spain]. Siglo Cero.

[20] Ball S., Holland T., Huppert F.A., Treppner P., Dodd K. (2006). CAMDEX-DS: The Cambridge Examination for Mental Disorders of Older People with Down’s Syndrome and Others with Intellectual Disabilities.

[21] Esteba-Castillo S., Novell I Alsina R., Vilà i Alsina M., Ribas i Vidal N. (2013). Prueba de Exploración CAMBRIDGE para la valoración de los Trastornos Mentales en Adultos con Síndrome de Down o con Discapacidad Intelectual [CAMBRIDGE Screening Test for the Assessment of Mental Disorders in Adults with Down Syndrome or Intellectual Disabilities].

[22] Cohen J. (1988). Statistical Power Analysis for the Behavioral Sciences.

[23] Navas P., Uhlmann S., Berasátegui A. (2014). Envejecimiento Activo y Discapacidad Intelectual [Active Ageing and Intellectual Disability].

[24] Manca R., De Marco M., Venneri A. (2020). The Impact of COVID-19 Infection and Enforced Prolonged Social Isolation on Neuropsychiatric Symptoms in Older Adults With and Without Dementia: A Review. Front. Psychiatry.

[25] Mok V.C., Pendlebury S., Wong A., Alladi S., Au L., Bath P.M., Biessels G.J., Chen C., Cordonnier C., Dichgans M. (2020). Tackling challenges in care of Alzheimer’s disease and other dementias amid the COVID-19 pandemic, now and in the future. Alzheimer’s Dement..

[26] Cuadrado M.C., Alonso M.V., Macho P.N., Torres S.M., González A.M.A. (2021). Impacto de la COVID-19 en las personas con discapacidades intelectuales y del desarrollo, sus familiares y los profesionales y organizaciones de apoyo [Impact of COVID-19 on people with intellectual and developmental disabilities, their families, support professionals and organizations]. Siglo Cero.

[27] Van Der Putten A., Vlaskamp C. (2011). Day Services for People With Profound Intellectual and Multiple Disabilities: An Analysis of Thematically Organized Activities. J. Policy Pr. Intellect. Disabil..

[28] Vlaskamp C., Hiemstra S.J., Wiersma L.A., Zijlstra B.J.H. (2007). Extent, Duration, and Content of Day Services? Activities for Persons With Profound Intellectual and Multiple Disabilities. J. Policy Pr. Intellect. Disabil..

[29] Barquín-Cuervo R., Medina-Gómez M.B., de Albéniz-Garrote M.B.M.-G.Y.G.P. (2018). El Uso de Estrategias de Afrontamiento del Estrés en Personas con Discapacidad Intelectual [Coping strategies among people with intellectual disability]. Psychosoc. Interv..

[30] Mendizabal M.R.L. (2018). Envejecimiento activo: Un cambio de paradigma sobre el envejecimiento y la vejez / Active Aging: A change of paradigm on aging and old age. Aula Abierta.

[31] Gómez-Marí I., Lacruz-Pérez I. (2021). Superando barreras en tiempos de pandemia. Discapacidad intelectual, familias y COVID-19 [Overcoming barriers in pandemic time: Intellectual disability, families and COVID-19]. Quaderns Digitals Rev. Nuevas Tecnol. Soc..

[32] Gibbons R.D., Hedeker D., DuToit S. (2010). Advances in Analysis of Longitudinal Data. Annu. Rev. Clin. Psychol..

[33] García J.D., Macho P.N. (2018). Deterioro cognitivo y trastorno neurodegenerativo en personas con discapacidad intelectual [Cognitive impairment and neurodegenerative disorder in people with intellectual disability]. Siglo Cero.

[34] Muñiz J. (2018). Introducción a la Psicometría. Teoría clásica y TRI [Introduction to Psychometry. Clasical theory and IRT].

